# Economic analysis of the costs associated with Hidradenitis suppurativa at a German University Hospital

**DOI:** 10.1371/journal.pone.0255560

**Published:** 2021-08-04

**Authors:** Verena Gerlinde Frings, Oliver Schöffski, Matthias Goebeler, Dagmar Presser

**Affiliations:** 1 Department of Dermatology, Venereology and Allergology, University Hospital Würzburg, Würzburg, Bavaria, Germany; 2 Chair of Health Management, School of Business, Economics and Society, Friedrich-Alexander-University Erlangen-Nürnberg, Nürnberg, Bavaria, Germany; University of Birmingham, UNITED KINGDOM

## Abstract

**Background and objectives:**

Hidradenitis suppurativa (HS) significantly affects the patient`s quality of life and leads to multiple medical consultations. Aim of this study was to assess the utilization of medical care of HS patients.

**Patients and methods:**

All patients presenting in 2017 for an outpatient, day patient and / or inpatient treatment with leading claim type HS at the Department of Dermatology, University Hospital Würzburg, were included. Primary outcome was the economic burden of HS patients, measured by resource utilization in €.

**Results:**

The largest share of the direct medical costs for HS were the inpatient costs with a leading surgical diagnosis-related group (DRG). Antiseptics were the predominant topical prescription. While doxycycline was the most frequently prescribed systemic therapy, adalimumab was the main cost driver. The difference between in-patient (€ 110.25) and outpatient (€ 26.34) direct non-medical costs was statistically significant (p < 0.001). With regards to indirect medical costs, a statistically significantly higher loss of gross value added (inpatient mean € 1,827.00; outpatient mean € 203.00) and loss of production (inpatient mean € 1,026.00; outpatient mean € 228.00) could be noted (p < 0.001), respectively.

**Conclusions:**

The present study on disease-specific costs of HS confirms that the hospital care of patients with this disease is cost-intensive. However, the primary goal of physicians is not and should not be to save costs regarding their patients`treatment, but rather the premise to utilize the existing resources as efficient as possible. Reducing the use of costly therapeutics and inpatient stays therefore requires more effective therapy options with an improved cost-benefit profile.

## Introduction

The interdisciplinary field of health economic research deals with the scarcity of resources in the health care system and their rational use [1]. By transparently assessing the value-for-money ratio of an intervention, an efficient use of scarce monetary resources is sought [2]. The primary goal is not to save costs in the healthcare sector, but rather the premise to utilize the existing resources as efficiently as possible [1].

Hidradenitis suppurativa (HS; also known as acne inversa) is a chronic inflammatory skin disease characterized by recurrent abscesses and sinus tract formation in the inverse areas of the body (including the armpits and groins) [3–5]. The prevalence of HS is estimated between 0.05% and 4% [6, 7]. However, it may be assumed that insufficient information is provided on the true prevalence of HS due to a low level of awareness and correspondingly reduced diagnosis of this disease [7]. Due to the relatively high number of affected persons, the chronic course of HS [8], and the association with comorbid diseases such as diabetes mellitus, obesity, chronic inflammatory bowel diseases (IBD), cardiovascular diseases and psychiatric disorders [8–14], the impact of this disease on the use of the health care system is significant. Aim of the present study was to investigate the attributable economic consequences of HS using a bottom-up-based methodology analyzing data of the HS clinic at the Department of Dermatology, Venereology and Allergology, University Hospital Würzburg, Germany.

## Patients and methods

### Study population

In May 2018 data from patients treated at the Department of Dermatology, Venereology and Allergology, University Hospital Würzburg, between January 1, 2017 and December 31, 2017 were collected and analyzed retrospectively. These data referred to patients presenting for an outpatient, day patient and / or inpatient treatment due to HS. Patients were either seen by a registrar or a specialist who also assessed disease severity and the impact of HS on quality of life. Surgically treated inpatients all underwent radical excision of the affected skin areas. Data were extracted from the hospital`s digital information system (SAP^®^, SAP SE Walldorf, Germany) and analyzed anonymously in a retrospective setting. No identifiable health information was included in this work. Accordingly, no further ethics vote was required (statement #20210209 01 by the Ethics Committee of the University of Würzburg). All underlying data are provided in the supplement (S1 Dataset).

### Assessment of disease severity

The most common score used to assess severity of HS is the Hurley staging [15] that allows a static but not a dynamic assessment of the disease. Stage (I) is characterized by single or multiple abscesses without sinus tracts or cicatrization; stage (II) by single or multiple recurrent abscesses with sinus tracts and cicatrization, widely separated; and stage (III) by diffuse involvement or multiple connected sinus tracts and abscesses.

### Dermatology life quality index

To assess the impact of HS on quality of life, the Dermatology Life Quality Index (DLQI) has been used [16]. The DLQI is derived from a validated 10-item questionnaire which addresses symptoms and the effect of the skin diseases on daily activities, leisure / sport, work / school as well as interpersonal relationships, and also takes treatment / therapy into account. The maximum score is 30, with 0 indicating a minimum and 30 a maximum of impairment in the patient’s quality of life [16].

### The German health care system

The health care system in Germany comprises three areas: outpatient care, the hospital sector and outpatient / inpatient rehabilitation facilities. It is based on the four basic principles of

(a) Compulsory insurance: All citizens are obliged to statutory health insurance. Those who earn above a certain limit can take out private health insurance.
(b) Financing of contributions: Health care is financed by contributions from citizens with health insurance, employers, and by subsidies from fiscal revenues.
(c) Principle of solidarity: All those insured under statutory health insurance jointly bear the risk of the costs arising from an illness.
(d) Principle of self-administration: The state specifies the framework conditions for medical care; further organization and financing is the responsibility of the so-called self-administration in the health system [1, 2].

### Disease-related costs

The analysis of disease-related costs was employed from the perspective of the statutory health insurance and was carried out on the basis of retrospective accounting data of the University Hospital Würzburg for the year 2017. Patients with a main diagnosis of HS were identified using ICD-10 codes.

Since we aimed for the clearest possible approach, there is no further elaboration of manufacturer discounts, pharmacy discounts and rebate agreements between the pharmaceutical industry and health insurances. Costs were classified into direct medical, direct non-medical and indirect health care costs, according to common economic analysis forms from the societal perspective [17].

I. Direct medical costs include the costs of outpatient, day-care or inpatient treatment and treatment cost. Treatment costs were assessed in accordance with the 2017 DRG (Diagnosis-Related Groups) and EBM (Standard Assessment /”*Einheitlicher Bewertungsmaßstab*”) guidelines. Drug costs were calculated according to the ABDAMED database (as of Dec 15, 2018) as if all patients were covered by statutory health care. The costs of systemic therapies has been calculated based on dosages for HS according to the European S1 guidelines and treatment regimens commonly used in clinical practice [18]. It was searched for both commercial and generic names. Treatment regimens were optimized for costs if several brands were available. Non-prescription drugs or wound dressings are not listed by costs in the ABDAMED database, so for this purpose, the currently lowest price was determined through an online publicly available drug search database [19].

II. Direct non-medical costs (such as travel costs to the hospital, the costs of renovation work in a patient’s house due to a disability caused by the illness or the costs incurred through the use of supporting services) related in our cohort only to expenses for travelling to the University Hospital Würzburg and were calculated as one-way distance (in km) per consultation multiplied by € 0.30.

III. Indirect costs due to incapacity to work were calculated on the basis of the classical human capital approach [20]. In order to assess the loss in added value, we referred to the production loss and the loss of gross value added per day of sick leave. The loss of production (average € 114) and the loss of gross value added (average € 203) per sick leave day were calculated on the basis of a national economic statement of the Federal Statistical Office of 2017 [21].

Costs were calculated for the prescribed maximum duration of treatment or for a maximum of 365 days in order to allow an adequate comparison and to reflect the long-term treatment for HS customary in clinical practice. If a topical or systemic therapy was only prescribed once during an outpatient visit and the patient consulted our specialty clinic again after three months, a pack size was calculated that averaged 12 weeks of therapy. For postoperatively formulated dressings, an average period of use of six weeks was estimated and calculated with an appropriate package size.

### Statistical analysis

Descriptive statistics are presented as mean ± standard deviation for numeric variables after testing for normality using Shapiro Wilk test. Non-normally distributed monetary variables are depicted as median and quartiles (min, max; Q25, Q75). As for the categorical variables "sex", “Hurley stage”, and "employment" the chi-square test was applied and as for the metric variable "age" an independent *t*-test. With regard to cost analysis, the indication of the mean and the standard deviation was omitted, due to a large degree of cluster skewness. Using logarithmic values the calculation still had a cluster of extreme values so that the significance of group differences for non-Gaussian distribution was tested using Mann-Whitney rank statistics with post-hoc Bonferroni correction and considered significant at p ≤ 0.05. For statistical analysis Microsoft Excel (version 16.0.8431.2110, Microsoft Corporation) and SPSS for Windows (version 24.0, Statistical Package for Social Sciences, SPSS Inc., Chicago, IL, USA) were used.

## Results

### Demographic and clinical characteristics

The evaluation period between 01/01/2017 and 31/12/2017 included 340 individual cases (♂ = 136, ♀ = 204, 38.7 ± 11.6 years; Table 1) with a diagnosis of HS. These cases were categorized according to their leading claim-type (inpatient, outpatient or day-treatment). Further sociodemographic data and clinical characteristics are shown in Table 1. There were no statistically significant differences in terms of sex, age distribution, employment, duration of disease or DLQI when comparing outpatient and inpatient cases, however, inpatients usually had a higher disease severity according to Hurley stage (Table 1, p < 0.001).

**Table 1:** Sociodemographic data and clinical characteristics of the study population.

Variable	Leading claim type			p value
	all patients (n = 340)	inpatients (n = 101)	Outpatients (n = 239)	
**Sex**	f = 204 (60%)	f = 67 (66%)	f = 137 (57%)	0.153
	m = 136 (40%)	m = 34 (34%)	m = 102 (43%)	
**Age in years** (mean±SD)	n = 340 (38.7 ± 11.6)	n = 101 (40.5 ± 12.6)	n = 239 (37.9 ± 11.1)	0.067
**Employed**	n = 251	n = 79	n = 172	0.325
	yes: 199 (79%)	yes: 59 (75%)	yes: 140 (81%)	
	no: 52 (21%)	no: 20 (25%)	no: 32 (19%)	
**Duration of illness in years** (mean±SD)	n = 340 (11.9 ± 8.9)	n = 101 (12.7 ± 9.6)	n = 239 (11.6 ± 8.5)	0.408
**DLQI** (mean±SD)	n = 237	n = 72	n = 165	0.445
	(11.3 ± 8.1)	(11.9 ± 8.1)	(11.0 ± 8.1)	
	0–10: 49%	0–10: 47%	0–10: 50%	
	11–20: 36%	11–20: 36%	11–20: 36%	
	21–30: 15%	21–30: 17%	21–30: 14%	
**Hurley stage**	n = 337	n = 101	n = 236	<0.001
	I: 27 (8%)	I: 1 (1%)	I: 26 (11%)	
	II: 115 (34%)	II: 26 (26%)	II: 89 (38%)	
	III: 195 (58%)	III: 74 (73%)	III: 121 (51%)	

n = number, f = female, m = male, SD = standard deviation, DLQI = dermatology life quality index.

### Treatment-related costs of Hidradenitis suppurativa

#### Direct medical costs

Direct medical costs consisted of hospital treatment (inpatient, day-care and / or outpatient; Table 2) and the prescription of appropriate therapeutics during outpatient treatment. The DRG for radical surgical intervention in HS patients defined the direct medical costs in the inpatient setting (ranging from € 2,024.18 to € 4,636.69 according to localization, wound closure type, and number of affected skin areas). A day-care claim was charged € 275.44. Pre- and post-treatment (5 days prior to and 14 days after inpatient treatment) are included in the given DRG. In the first and second quarters of 2017, the allowance of the hospital`s outpatient treatment amounted € 107.64 and € 147.12 in the third and fourth quarters.

**Table 2:** Direct medical costs.

Variable	Leading claim type		
	all patients (n = 340)	Inpatients (n = 101)	outpatients (n = 239)
	Total (mean ± SD)	Total (mean ± SD)	Total (mean ± SD)
**Visits**			
• inpatient	122 (0.4 ± 0.7)	122 (1.2 ± 0.8)	NA
• day-care	11 (0.0 ± 0.2)	11 (0.1 ± 0.3)	NA
• outpatient	598 (1.8 ± 1.0)	242 (2.4 ± 1.1)	356 (1.5 ± 0.7)
**Accounting** (€)			
• inpatient	417,840.53 (1,228.94 ± 2,729.29)	417,840.53 (4,137.03 ± 3,611.80)	NA
• day-care	4,142.76 (12.18 ± 79.63)	4,142.76 (41.02 ± 142.00)	NA
• outpatient	75,017.36 (220.64 ± 124.86)	29,774.52 (294.80 ± 153.69)	45,242.84 (189.30 ± 94.28)
**Total allowances** (€)	497,000.65 (1,461.77 ± 2,770.50)	451,757.81 (4,472.85 ± 3,594.41)	45,242.84 (189.30 ± 94.28)

Direct medical costs include the costs of outpatient, day-care or inpatient treatment and treatment cost. Treatment and drug costs were calculated as if all patients were covered by statutory health care. n = number, SD = standard deviation, NA = not applicable.

Referring to the leading claim type, the inpatient cohort (n = 101) accounted for 122 inpatient stays with a total of 753 (6.4 ± 2.8) days of hospitalization, 11 day-care treatments and an additional 242 outpatient claims with a total of 345 visits. Inpatient stays amounted with € 417,840.53, day-care treatment with € 4,142.76 and outpatient treatment with € 29,774.52 (Table 2).

For the outpatient cohort, 359 visits of 239 individuals were documented in 2017. Due to the quarterly accounting this resulted in 356 outpatient cases. The allowance for leading outpatient claim type totaled up to € 45,242.84 (Table 2).

#### Direct non-medical costs

The direct non-medical costs consisted solely of the costs for the travelled distance between the place of residence and the Department of Dermatology (Table 3). The inpatient range was slightly larger with an average journey distance of 121.0 km ± 93.3 km compared to 87.6 km ± 73.5 km in the outpatient group. For the one-way distance, the median cost factor was € 32.70 in the inpatient and € 20.55 in the outpatient group per patient per visit. Extrapolated to all visits or journeys to the University Hospital Würzburg, this amounted to € 110.25 for each inpatient and to € 26.34 for each outpatient for all visits in 2017. The difference between out- and inpatient costs was statistically significant (p < 0.001).

**Table 3:** Direct non-medical costs.

Variable	Leading claim type			p value
	All patients (n = 340)	Inpatients (n = 101)	Outpatients (n = 239)	
**One-way distance in** km (mean ± SD)	33,160.1 (97.5 ± 81.4)	12,223.7 (121.0 ± 93.3 km)	20,936.4 km (87.6 km ± 73.5 km)	
**Total visits** (mean ± SD)	840 (2.5 ± 2.1)	481 (4.8 ± 2.5)	359 (1.5 ± 0.8)	
**Travel costs per visit** in €, Median (Min;Max)[Q25;Q75]	22.52 (0.90;157.50) [11.78;41.48]	32.70 (0.90;157.50) [14.13;49.50]	20.55 (0.90;113.10) [9.93;36.45]	
**Total travel costs** in €, Median (Min;Max) [Q25;Q75]	38.85 (0.90;553.50) [17.50;91.44]	110.25 (3.60;553.50) [52.08;244.80]	26.34 (0.90;249.00) [13.26;52.50]	<0.001

Direct non-medical costs related mainly to expenses for travelling due to treatment at the University Hospital Würzburg. SD = standard deviation. n = number, Min = minimum, Max = maximum, Q25 = 1st quartile, Q75 = 2nd quartile.

#### Indirect medical costs

Indirect medical costs were assessed by means of loss of production (estimated based on labor costs) and loss of gross value added (estimated loss of labor productivity) due to hospital treatment. For both methods, the outage and associated costs are higher for the inpatients than for the outpatients. Accordingly, both calculation methods showed a statistically significantly higher monetary loss in case of inpatient treatment (Table 4).

**Table 4:** Indirect medical costs.

Variable	Leading claim type			p value
	All patients (n = 340)	Inpatients (n = 101)	Outpatients (n = 239)	
**Work absenteeism** in days (mean ± SD)	1417 (4.2 ± 5.8)	1058 (10.5 ± 7.4)	359 (1.5 ± 0.8)	<0.001
**Loss of production** in €, Median (Min;Max) [Q25;Q75]	228.00 (114.00;3,192.00) [114.00;570.00]	1,026.00 (114.00;3,192.00) [684.00;1,254.00]	114.00 (114.00;570.00) [114.00;228.00]	<0.001
**Loss of gross value added** in €, Median (Min;Max) [Q25;Q75]	406.00 (203.00;5,684.00) [203.00;1,015.00]	1,827.00 (203.00;5,684.00) [1,218.00;2,223.00]	203.00 (203.00;1,015.00) [203.00;406.00]	<0.001

Indirect costs were calculated on the basis of the classical human capital approach with loss of production and loss of gross value added due to hospital treatment per day of sick leave. n = number, SD = standard deviation. Min = minimum, Max = maximum, Q25 = 1st quartile, Q75 = 2nd quartile.

#### The costs for the patient and the statutory health insurance

Travelling to the hospital and paying for the prescribed local and systemic therapies (deductible expenses) resulted in total costs of € 310.32 per patient in the inpatient and € 67.78 in the outpatient group. This meant a statistically significant additional monetary burden for patients who needed hospitalization. Despite the higher number of cases in the outpatient group, the inpatient cohort was significantly more cost-intensive, mainly due to significantly higher costs for the statutory health insurance of the inpatient stay (p < 0.001) but also due to higher costs of postoperative topicals and wound dressings (p < 0.001) and higher travel costs (p < 0.001) due to further follow-up appointments. The costs for the statutory health insurance resulted in € 3,300.17 per inpatient compared to € 147.20 per leading outpatient claim type (p < 0.001). The prescribing of topical medications, systemic therapies or wound dressings did not lead to a statistically significant increased monetary burden in one or another group. However, higher mean costs for systemic therapies incurred both for the statutory health insurance and the patients regarding inpatient claims (inpatients: statutory health insurance € 2,261.69 ± 9,754.74, patient`s contribution € 8.76±22.37; outpatients: statutory health insurance € 1,262.13±7,145.77, patient`s contribution € 5.86±14.85). Altogether, the statutory health insurance showed a higher mean monetary burden for patients with leading surgical claim type (p < 0.001) (Table 5).

**Table 5:** Patient`s contribution and costs of the statutory health insurance.

Variable	Leading claim type			p value
	All patients (n = 340) Median in € (Min;Max) [Q25;Q75]	Inpatients (n = 101) Median in € (Min;Max) [Q25;Q75]	Outpatients (n = 239) Median in € (Min;Max) [Q25;Q75]	
**Patient`s contribution**				
○ distance travelled	38.85 (0.90;553.50) [17.50;91.44]	110.25 (3.60;553.50) [52.08;244.80]	26.34 (0.90;249.00) [13.26;52.50]	<0.001
○ hospital stay	0.00 (0;340.00) [0;40.00]	60.00 (0;340.00) [50.00;80.00]	NA	NA
○ treatments	46.44 (0;426.13) [31.22;102.43]	120.31 (3.80;426.13) [75.49;188.89]	36.44 (0;325.96) [28.80;58.75]	<0.001
• Topical	36.22 (0;184.88) [27.68;67.44]	72.44 (3.80;184.88) [40.24;112.46]	32.60 (0;177.66) [22.38;44.76]	<0.001
• systemic	0.00 (0;100.00) [0;10.00]	0.00 (0;100.00) [0;10.00]	0.00 (0;100.00) [0;10.00]	0.353
• wound dressings	0.00 (0;297.69) [0;21.02]	42.04 (0;297.69) [21.02;44.01]	0.00 (0;251.32) [0;0]	<0.001
**Total patient`s contribution**	105.16 (6.60;1,123.84) [54.85;218.53]	310.32 (53.52;1,123.84) [232.86;479.32]	67.78 (6.60;574.96) [49.65;116.11]	<0.001
**Health insurance`s contribution**				
○ distance travelled	NA	NA	NA	NA
○ hospital stays	254.84 (107.64;28,683.88) [147.20;2,954.30]	3,300.17 (462.37;28,383.88) [3,112.84;3,783.93]	147.20 (107.64;509.68) [107.64;254.84]	<0.001
○ treatments	20.91 (0;44,846.71) [0;42.08]	0.00 (0; 44,846.71) [0;42.08]	20.91 (0;44,798.79) [0;42.08]	0.170
• topical	0.00 (0;143.50) [0;32.23]	0.00 (0;118.23) [0;18.50]	18.50 (0;143.50) [0;37.00]	0.004
• systemic	0.00 (0;44,846.71) [0;23.58]	0.00 (0;44,846.71) [0;23.58]	0.00 (0;44,780.29) [0;23.58]	0.353
• wound dressings	NA	NA	NA	NA
**Total health insurance`s contribution**	275.75 (107.64;51,670.18) [147.20;3,197.01]	3,397.06 (462.37;51,670.18) [3,209.84;5,908.37]	170.78 (107.64;45,200.57) [134.58;294.25]	<0.001

In order to differentiate between the costs that are covered by the statutory health insurance and the costs that have to be borne by the patients themselves, this table provides the total costs of allowances / deductibles that the patient or the health insurance company has with regard to travel costs, hospitalization and treatment modalities. SD = standard deviation. n = number, Min = minimum, Max = maximum, Q25 = 1st quartile, Q75 = 2nd quartile, NA = not applicable.

With regard to topical and systemic therapeutics and wound dressings, antiseptics were the predominant prescription in both the out- and inpatient group. While doxycycline was the most frequently prescribed systemic therapy, adalimumab (Humira^®^) was the main cost driver with a maximum of up to € 44,846.91 per patient treated. Dressing materials were more frequently prescribed in the in- rather than in the outpatient group and thus contributed significantly to the cost development in this cohort (Table 5).

#### Total costs

The total medical expenses of the patients treated in 2017 at the Department of Dermatology, Venereology and Allergology of the University Hospital Würzburg amount to median € 545.99 per patient. This consists primarily of direct medical costs and subsequently of indirect and direct non-medical costs. Here, too, the inpatient sector is statistically significant (p < 0.001) cost-pushing with a total of € 4,493.89 per patient compared to the outpatient collective with total costs of € 333.02 per patient. This can be explained by the higher direct medical costs (Table 6). Overall, the diagnosis "Hidradenitis suppurativa" resulted in total costs of € 1,175,184.90 at our department in 2017.

**Table 6:** Total costs.

Variable	Leading claim type			p value
	All patients (n = 340) Median in € (Min;Max) [Q25;Q75]	Inpatients (n = 101) Median in € (Min;Max) [Q25;Q75]	Outpatients (n = 239) Median in € (Min;Max) [Q25;Q75]	
**Direct medical costs**	331.57 (107.64;51,929.07) [178.42;3,280.09]	3,550.87 (466.75;51,929.07) [3,298.06;6,090.05]	226.35 (107.64;45,487.29) [164.77;345.91]	<0.001
**Direct non-medical costs**	38.85 (0.90;553.50) [17.50;91.44]	110.25 (3.60;553.50) [52.08;244.80]	26.34 (0.90;249.00) [13.26;52.50]	<0.001
**Indirect costs**	228.00 (11.,00;3,192.00) [114.00;570.00]	1,026.00 (114.00;3,192.00) [684.00;1,254.00]	114.00 (114.00;560.00) [114.00;224.00]	<0.001
**Total costs**	545.99 (114.24;52,180.20) [287.68;3,641.09]	4,493.89 (743.89;52,180.20) [3,674.13;6,759.49]	335.02 (114.24;45,763.47) [259.04;579.82]	<0.001

Direct medical, direct non-medical and indirect costs comprise the total costs of HS, highlighting the difference between outpatients and inpatients. SD = standard deviation. n = number, Min = minimum, Max = maximum, Q25 = 1st quartile, Q75 = 2nd quartile.

## Discussion

With regard to the economic burden of HS for the German health care system, neither studies on out- or inpatient services nor therapy costs have been published to date. For the first time, the current study summarizes medical costs of HS in Germany exemplified by the Department of Dermatology, Venereology and Allergology of the University Hospital Würzburg. However, there is already comparable data available on the economic situation regarding this skin disease from the United States [13, 22–24] and from a single British cohort study [25].

A cohort study for cost-of-illness (COI) identification by Kirby and colleagues [22] showed that the largest proportion of the 3-year total cost of the studied HS group accounted for inpatient care (37.4%). In the cohort presented by us, inpatient treatment also proved to cause the highest costs. Interestingly, the mentioned US-cohort compared the costs inflicted by HS to a psoriasis group, another severe relapsing chronic dermatosis, where the largest proportion of the 3-year total cost accounted for drug costs (46.5%). Additionally, the proportion of patients who were hospitalized or used the emergency department in the HS cohort was higher than in the psoriasis group (p < 0.001, respectively). This study highlights that high-cost settings, such as emergency department and inpatient care, are used more frequently by patients with HS. In accordance, a prior study already showed a higher proportion of HS (5.1%) than psoriasis (1.6%) patients in need of hospitalization (p < 0.001) [24].

Another retrospective cohort study from the United States analyzed the relationship between HS and comorbidity, particularly irritable bowel disease (IBD), and the associated resource use [13]. Patients with IBD and HS had a statistically significantly longer hospital stay (5 vs. 4 days; p <0.001) and higher hospitalization costs ($ 13,272 vs. $ 12,237; p = 0.013) than patients with IBD alone. This underlines the extent to which HS increases the associated resource requirements. However, this also means that a much higher total resource requirement due to associated comorbidities such as obesity, diseases of the cardiovascular system, and metabolic disorders is certainly to be assumed in our HS patients.

The only cohort study on resource use due to HS in another European health care system focused on inpatients; outpatients treated with leading claim type HS were excluded [25]. This British study was able to report on 11,359 HS patients with 65,544 inpatient stays and 303,204 accompanying outpatient visits between 2007 and 2013. The investigated UK cohort accounted for an average cost of £ 2,027 (= approximately € 2,290) per patient per year. In comparison, total costs (direct medical, direct non-medical and indirect costs) were amounted at € 4,473.89 per patient with leading inpatient claim type in our study. Thus, highly differing costs can be expected for HS inpatient treatment in European countries, probably relating to differences in health care systems.

With regard to the costs incurred for therapeutics and radical surgical intervention for HS, the present study is the first in German-speaking countries. Concerning biologics approved for HS (such as adalimumab), treatment costs are certainly very high (total costs of € 529,536.35 in n = 13 compared to € 3,408.13 in n = 99 patients treated with doxycycline). The latter therapy is often given only for 12 weeks. Adalimumab, however, is used as a long-term therapeutic and has been calculated in the current study for the course of one year. As previously stated, a larger cohort study addressed, inter alia, the monetary expenses for pharmaceuticals in HS compared to psoriasis vulgaris and a skin healthy control group [22]. The cost of pharmaceuticals in the psoriasis vulgaris cohort was higher than in the HS group. However, it must be noted that this study was performed prior to the approval of the costly biologic adalimumab as first-line treatment of HS [26, 27], so that the higher treatment costs of psoriasis vulgaris have to be questioned and require further analysis. In addition, these treatment costs might be considerably lower due to the launch of biosimilars. The higher mean monetary burden for patients with leading surgical claim type in our study has to be interpreted with care, since adalimumab is usually more expensive but was less often administered at our department in 2017. This is because the common nonsurgical methods rarely result in a lasting cure and radical surgical treatment seems to be accompanied by lower recurrence rates [18, 28], even if a direct comparison has not yet been made. In addition, adalimumab seems to be effective only temporarily or in high dosages over a short period of time to condition for a surgical approach [18].

Another study [23] focused in particular on the indirect economic costs of HS. These patients had statistically significantly more sick days (18.4 vs. 7.7), higher annual indirect health care costs ($ 2,925 vs. $ 1,482) and lower annual incomes ($ 54,925 vs. $ 62,357) compared to the control group (p < 0.001, respectively). Our cohort also showed a higher loss of production or loss of gross value added. However, since no healthy control group was included in our study, the only conclusion for both methods of calculation to be made is that the absenteeism from work and the associated costs were on average higher in inpatients than in outpatients (p < 0.001). It should be noted that these outpatients were often on sick leave for a very long time prior to the inpatient stay and can—from our experience—usually be brought back to work after a post-inpatient rehabilitation period. Effective treatments for HS may therefore reduce this incremental indirect monetary burden.

A limitation of the present study is that only patients with HS were included and no comparison to another population such as patients with leading diagnosis psoriasis vulgaris took place. Moreover, a potential selection bias has to be noted, as the cohort studied here is dominated by patients with high disease severity (58% Hurley Stage III vs. 34% Hurley Stage II vs. 8% Hurley Stage I). This can certainly be explained by the Department of Dermatology, Venereology and Allergology of the University Hospital Würzburg being a specialty center for HS. High treatment costs might be related to disease activity, this could not be adequately analyzed in our patient group due to mainly patients in Hurley Stage III. In addition, disease severity and outcome according to the chosen treatment approach could not be analyzed adequately due to insufficient data. Furthermore, the commuting area of the University Hospital Würzburg is rather rural and large so that travel costs might be much higher than in major cities. It should also be mentioned critically that the present work deals only with patient data of one year. This is a relatively short period of time for a chronic relapsing disorder. Although multiple surgeries are often required for multiple affected locations, the present analysis may provide a distorted picture of medical expenses between inpatients and outpatients. By using a cost-benefit analysis, it could well be that inpatients and thus in our case radically surgically treated patients ultimately cause a lower economic burden for the healthcare system over their lifetime than outpatients with their often frequent visits for their chronic relapsing skin diseases. We hypothesize that this could be particularly true since radical surgical treatment tends to show lower recurrence rates than systems therapies used for HS [18, 28].

The main strength of the present study is that the diagnosis of HS in the present patient group was made by qualified dermatologists and, accordingly, the corresponding DRG assignment is reliable. In addition, both costs for pharmaceuticals and distance travelled to the hospital were recorded, so that a clear statement about direct medical and indirect costs could be made.

It should be noted that data on the medical and economic resource utilization of HS patients is internationally scarce and hardly exists at national levels. With regards to the prevalence of HS, the increasing reliability of diagnosis, the severity of the disease and the sometimes high-priced therapy options, it is essential to carry out further analyzes of the COI in HS.

## Conclusion

The present study on disease-specific costs of HS exemplified by the Department of Dermatology, University Hospital Würzburg, confirms—in accordance with prior studies comparing HS costs and medical care utilization to psoriasis cohorts—that the hospital care of HS patients is cost-intensive. Minimizing cost-intensive treatment modalities may lead to significant cost savings. However, the primary goal of physicians is not and should not be to save costs regarding their patients`treatment, but rather the premise to utilize the existing resources as efficient as possible. Reducing the use of costly therapeutics and inpatient treatment requires more effective therapy options with an improved cost-benefit profile. The current study draws attention to the fact that the costs of inpatient to outpatient treatment can and should only be compared very carefully. Patients treated in hospitals are usually more seriously ill than patients who can be managed on an outpatient basis. Moreover, (inpatient) radical surgical intervention is seen as a treatment approach with relatively low recurrence rates while cost-intensive medications such as adalimumab, which are used both for outpatients as mono-therapy as well as for preoperative reduction of inflammatory activity in patients undergoing surgery, is often not sustainably controling the disease. Real-life analysis of the medical costs in HS should consider this situation, which, however, is compounded by the fact that long-term outcome data of different treatment approaches is missing.

## Supporting information

S1 Dataset (XLSX)
