# Correction: Economic analysis of the costs associated with Hidradenitis suppurativa at a German University Hospital

**DOI:** 10.1371/journal.pone.0257481

**Published:** 2021-09-10

**Authors:** Verena Gerlinde Frings, Oliver Schöffski, Matthias Goebeler, Dagmar Presser

The following information is missing from the Funding statement: This publication was supported by the Open Access Publication Fund of the University of Wuerzburg.
